# Production and characterization of freeze‐dried banana slices pretreated with ascorbic acid and quince seed mucilage: Physical and functional properties

**DOI:** 10.1002/fsn3.1666

**Published:** 2020-05-28

**Authors:** Alireza Milani, Mohammad Jouki, Mohammad Rabbani

**Affiliations:** ^1^ Department of Food Science and Technology Faculty of Biological Sciences North Tehran Branch Islamic Azad University Tehran Iran; ^2^ Department of Chemistry North Tehran Branch Islamic Azad University Tehran Iran

**Keywords:** antibrowning agent, antioxidant activity, banana slices, freeze‐drying

## Abstract

The objective of this investigation was to illustrate the effects of quince seed mucilage (QSM) and ascorbic acid pretreatments to prevent the quality of freeze‐dried banana slices. The studied parameters were moisture content, antioxidant activity, total phenol, color properties, structural properties, and sensory evaluation. Both treatments were effective in protecting total phenolic content and antioxidant activity in dried banana slices (*P* ˂ .05). The control slices showed greater increase in browning index (BI) and greater decrease in lightness (L*) than pretreated dried samples. Ascorbic acid and QSM treatments can be effective in the control of the enzymatic browning along with maintaining the quality properties of banana chips. Therefore, using of immersion pretreatment with 0.25% QSM and 0.05% ascorbic acid is recommended to prevent enzymatic browning as well as maintain the quality of banana chips before the drying process.

## Graphical abstract

The studied parameters were moisture content, antioxidant activity, total phenol, color properties, structural properties and sensory evaluation. r decrease in lightness (L*) than pre‐treated dried samples. Ascorbic acid and QSM treatments can be effective in the control of the enzymatic browning along with preserving the quality properties of banana slices. Therefore, using of immersion pretreatment with 0.25% QSM and 0.05% ascorbic acid is recommended to prevent enzymatic browning as well as maintain the quality of banana slices prior to the drying process.

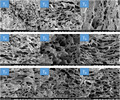

## 1 INTRODUCTION

Enzymatic browning is one of the most important issues in fresh‐cut fruit industry. It destroys the color and quality of the cut fruit and effects adversely on its quality (Alipoorfard, Jouki, & Tavakolipour, 2020; Golly et al., 2019; Sarpong et al., 2018). As illustrated by Lee and Eun (1999), browning is caused by the oxidation reaction of phenolic compounds through polyphenol oxidase (PPO). Subsequently, o‐quinone is formed and, as a result of polymerization, produces dark melanin pigments. In recent years, several solutions have been applied to prevent the enzymatic browning, like heat, lowering the pH of the fruit, the use of preservative chemicals, reducing the amount of oxygen present, or by adding some antibrowning compounds (Annese, Manzano, & Nicoli, 1997; Gorny, 1997; Sapers, Garzarella, & Pilizota, 1990; Sun, Lee, & Song, 2002).

Dipping treatment is one of the best ways that could be employed to inhibit enzymatic browning in fresh‐cut fruit (Pan, Shih, McHugh, & Hirschberg, 2008). Different compounds such as calcium chloride, citric acid, sodium ethanol, sodium chloride, carbonate, ascorbic acid, cysteine, cinnamic acid**,** ferulic acid, P‐coumaric acid, sinapic acid, alginate, gellan, and xanthan gum are used as antibrowning factor (Alipoorfard et al., 2020; Arias, González, Oria, & Lopez‐Buesa, 2007; Jiang, Fu, Zauberman, & Fuchs, 1999; Moreira, Cassani, Martín‐Belloso, & Soliva‐Fortuny, 2015; Nicoli, Anese, & Severini, 1993; Sharma & Rao, 2015; Yildiz, 2018). Therefore, it is reasonable to use an immersion pretreatment method to maintain the color of fresh‐cut fruits prior to the drying process.

Quince seed mucilage (QSM) is a set of a cellulosic fractions with a more hydrolyzed polysaccharides (Schmidt, 1844). As we previously mentioned, QSM is obtained from quince seeds when they are soaked in water and a transparent gel is formed around the seed (Jouki, Mortazavi, Yazdi, & Koocheki, 2014a). This antioxidant hydrocolloid is of interest as a gelling agent because of its unique colloidal properties, high viscosity, low production cost, and easy extraction (Jouki, Mortazavi, Yazdi, & Koocheki, 2014b).

Ascorbic acid has long been used in combination with other compounds to inhibit enzymatic browning of fruits (Ali et al., 2020; Rojas‐grau, Soliva‐Fortuny, & Martín‐Belloso, 2008; Sikora & Świeca, 2018; Soliva‐Fortuny, Grigelmo, Odriozola‐Serrano, Gorinstein, & Martín‐Belloso, 2001; Soliva‐Fortuny, Oms‐Oliu, & Martín‐Belloso, 2002; Zhao et al., 2019). Previously, the effect of substitution of 4‐hexylersorcinol, N‐acetylcysteine and glutathione on polyphenol oxidase (PPO) and peroxidase (POD) instead of ascorbic acid or in combination with ascorbic acid on fresh‐cut apple (Fuji variety) has been studied (Rojas‐grau et al., 2008). They showed that storage time increased PPO activity, while N‐acetylcysteine and glutathione inhibited enzyme activity. Gorny, Hess‐Pierce, Cifuentes, and Kader (2002) stated that the application of immersion solutions of cysteine, calcium lactate, and ascorbic acid increased the shelf life of pear slices by preventing the browning and tissue deterioration (*P* ˂ .05). As stated by Sikora and Świeca, (2018), ascorbic acid reduces enzymatic browning by inhibiting the activities of PPO and POD enzymes. As an effective inhibitor of enzymatic browning, it has capacity to reduce quinones to phenolic compounds before they can participate to pigment formation (McEvily et al., 1992).

To preserve food products like fruits and vegetables and prolong their shelf life, drying methods have been widely used. As reported by Bi et al. (2015), freeze‐drying is recognized as one of the most effective methods for decreasing moisture and inhibition of microbial growth and enzymatic browning. It has been employed to reduce the undesirable changes related to dried fruits (Alipoorfard et al., 2020; Pan et al., 2008), and considered as the best method due to low‐temperature and more maintenance of bioactive compounds in comparable with the thermal methods (Ratti, 2001). In recent years, freeze‐drying has been employed with other techniques and treatments to extend the shelf life of fruit slices. Based on our knowledge, no work is available on the effect of quince seed mucilage and ascorbic acid treatments on the quality parameters of freeze‐dried banana slices. So, current research aims to determine the qualitative parameters, antioxidant activity, dehydration, and browning index of pretreated freeze‐dried banana slices compared to freeze‐dried slices.

## 2 MATERIALS AND METHODS

### 2.1 Materials

All chemicals such as ascorbic acid, ethanol, methanol, and DPPH were purchased from the Sigma‐Aldrich company (Spain). Bananas and quince seed were prepared from a local market in Tehran (Iran).

### 2.2 Extraction of mucilage from quince seed

A sequential process was employed to extract mucilage from quince seeds using the technique explained by Jouki, Yazdi, Mortazavi, and Koocheki (2014c).

### 2.3 Sample and pretreatment preparation

Bananas (cv. Cavendish) with uniform size and color were selected, peeled, and cut into 30 equal slices (3 mm thickness) using a sharp steel knife. The fresh‐cut banana slices were immersed in solutions (Table 1) for 1 min, then drained and placed in plates, and finally stored at −18°C for 6 hr. Subsequently, drying was employed to dry the thin banana slices.

**TABLE 1:** The changes in the color parameters (a*, b*, and L*) and browning index (BI) in the freeze‐dried banana slices^a^

Treatments	L (Mean ± *SD*)	a (Mean ± *SD*)	b (Mean ± *SD*)	BI (Mean ± *SD*)
T_1_ (QSM 0.00% + AA 0.00%)	74.296 ± 0.133e	0.072 ± 0.030a	10.064 ± 0.904a	14.98 ± 1.07a
T_2_ (QSM 0.00% + AA 0.05%)	74.625 ± 0.939e	−0.884 ± 0.415b	8.595 ± 0.060b	12.28 ± 0.23ab
T_3_ (QSM 0.00% + AA 0.10%)	78.642 ± 0.534ab	−2.826 ± 0.059c	5.573 ± 0.744d	5.32 ± 0.55cd
T_4_ (QSM 0.25% + AA 0.00%)	76.296 ± 0.274d	−2.037 ± 0.424c	7.111 ± 0.835c	8.85 ± 2.04bc
T_5_ (QSM 0.25% + AA 0.05%)	76.609 ± 0.734cd	−2.302 ± 0.023c	3.918 ± 0.921e	3.79 ± 1.17d
T_6_ (QSM 0.25% + AA 0.10%)	77.720 ± 0.114bc	−2.552 ± 0.484c	3.791 ± 0.721e	5.91 ± 3.40cd
T_7_ (QSM 0.50% + AA 0.00%)	79.255 ± 0.933a	−2.316 ± 0.723c	4.489 ± 0.042de	5.13 ± 0.71cd
T_8_ (QSM 0.50% + AA 0.05%)	77.078 ± 0.276cd	−2.022 ± 0.146c	4.518 ± 0.000de	6.66 ± 1.95cd
T_9_ (QSM 0.50% + AA 0.10%)	77.806 ± 0.302bc	−2.456 ± 0.231c	5.053 ± 0.105de	5.74 ± 0.04cd

^a^ Means within each column followed by different letters (a‐e) show significant different (*p* < .05) between treatments.

### 2.4 Drying process

The banana slices were dried using an Armfield vacuum freeze‐dryer (Armfield FT‐33, Ltd.) according to the method of Gębczyński, Skoczeń‐Słupska, and Kur (2017). Briefly, the banana slices (36 slices per each run) were freeze‐dried for a period of 24 hr at 23–45 Pa with the condenser temperature at −49°C. The studied parameters after drying process were moisture content, antioxidant activity loss, color properties, structural properties, and sensory evaluation.

### 2.5 Determination of moisture content

The moisture content of freeze‐dried banana slices was determined in a vacuum oven at 70°C for 48 hr, according to AOAC (2000). The digital balance used for the measurement had an accuracy of 0.001 g (JKH‐500). The moisture content calculated using Equation (1):

(1) $$\text{MC}=(M_{b}-M_{a}/M_{b})\times 100$$

where M_b_ is the moisture content of dried banana slices before drying in oven and M_a_ is the moisture content after oven.

### 2.6 DPPH radical scavenging activity of Banana slices

The antioxidant activity of the dried banana chips was evaluated by method of the free radical scavenging effect on 2, 2‐diphenyl‐1‐picrylhydrazyl (DPPH) radical described by Brand‐Williams, Cuvelier, and Berset (1995). Briefly, 20 g of each banana slice was extracted using an Ultra Turrax homogenizer (IKA T25‐Digital) in 200 ml methanol: water (80:20) solvent mixture. Afterward, the mixture was centrifuged at 10,000 × *g* for 15 min at 5°C by an Ultra Centrifuge (Sigma 8K) and 0.1 ml of the supernatant was added to 3.9 ml of methanolic DPPH solution (at a concentration of 0.1 mM). Then, the obtained mixture was shaken and placed in a dark place for half an hour at room temperature. Absorption of the samples against a blank of methanol without the DPPH reagent was determined at 517 nm (using a PerkinElmer Spectrophotometer). The antioxidant activity of dried banana slices was expressed as µmol TE (trolox equivalents)/ g based on dry weight.

### 2.7 Total phenolic content (TPC)

Total phenolic content (TPC) was determined by Folin–Ciocalteu reagent (FCR) method as cited by Lee et al. (2004). Briefly, 20 g of each banana slice was extracted in 200 ml methanol: water (80:20) solvent mixture using Ultra Turrax homogenizer. Then, the mixture was centrifuged at 10,000 × *g* for 15 min and 0.1 ml of the supernatant was reacted with 0.2 ml FCR, comprehensively vortexed, and 0.8 ml (700 mmol/L) Na_2_CO_3_ was added to the assay mixture. Then, the mixture was placed in a dark place for an hour at room temperature. After 1‐hr incubation, UV–Visible Spectrophotometer at 765 nm was used to measure the absorbance of banana extracts. Gallic acid was applied as the standard, and the results were obtained based on a standard curve equation of gallic acid (0.05–0.75 mM). Experiment was performed in four replications, and the mean of replicates was expressed as mg of gallic acid equivalent (GAE) per gram based on dry weight.

### 2.8 Color and browning index (BI)

After freeze‐drying, color values of banana slices were measured by using Konica Minolta Colorimeter. Color was measured using the CIE L (lightness), a (redness), and b (yellowness) coordinates. For each sample, color values (L, a, and b) were read 6 times at different locations on the banana slice surface. As described by Perez‐Gago et al. (2006), the browning index (BI) was estimated with the following Equations 2 and 3:

(2) BI=(100×(x-0.31)/0.172)

(3) x=(a+1.75L/5.645L+a-3.012b)

### 2.9 Microstructure analysis

Scanning electron microscopy, SEM (AIS‐2100), was applied to analyze the internal structure of freeze‐dried banana slices after dipping pretreatment according to the method of Jouki et al. (2014a). The samples were cut by liquid nitrogen and mounted on the holder by aluminum tape. Then, the samples were coated by gold in sputter coater (BAL‐TEC SCD, Liechtenstein) for 40 s at 20 mA. All samples were examined using an accelerating beam at a voltage of 6.0 kV. Magnification of 250× was used.

### 2.10 Sensory evaluation

Sensorial tests were performed by comparison with 15 semitrained panelists (including 8 women and 7 men, food science specialists, age 28–42) in standardized individual cabins possessing adequate illumination. The panelists were trained to evaluate sensorial parameters of dried banana slices. The scores (1 = dislike very much, 2 = dislike a little, 3 = neither like nor dislike, 4 = like a little, and 5 = like very much) were given by the panelists (Meilgaard et al., 1999).

### 2.11 Statistical analysis

All data were used to the analysis of variance using SPSS software (version 21.0) and were presented as mean values with standard deviations. All experiments were performed in four replications, and Duncan's multiple range tests were used to compare the differences among means.

## 3 RESULTS AND DISCUSSION

### 3.1 Moisture content of freeze‐dried banana slices

**FIGURE 1:**
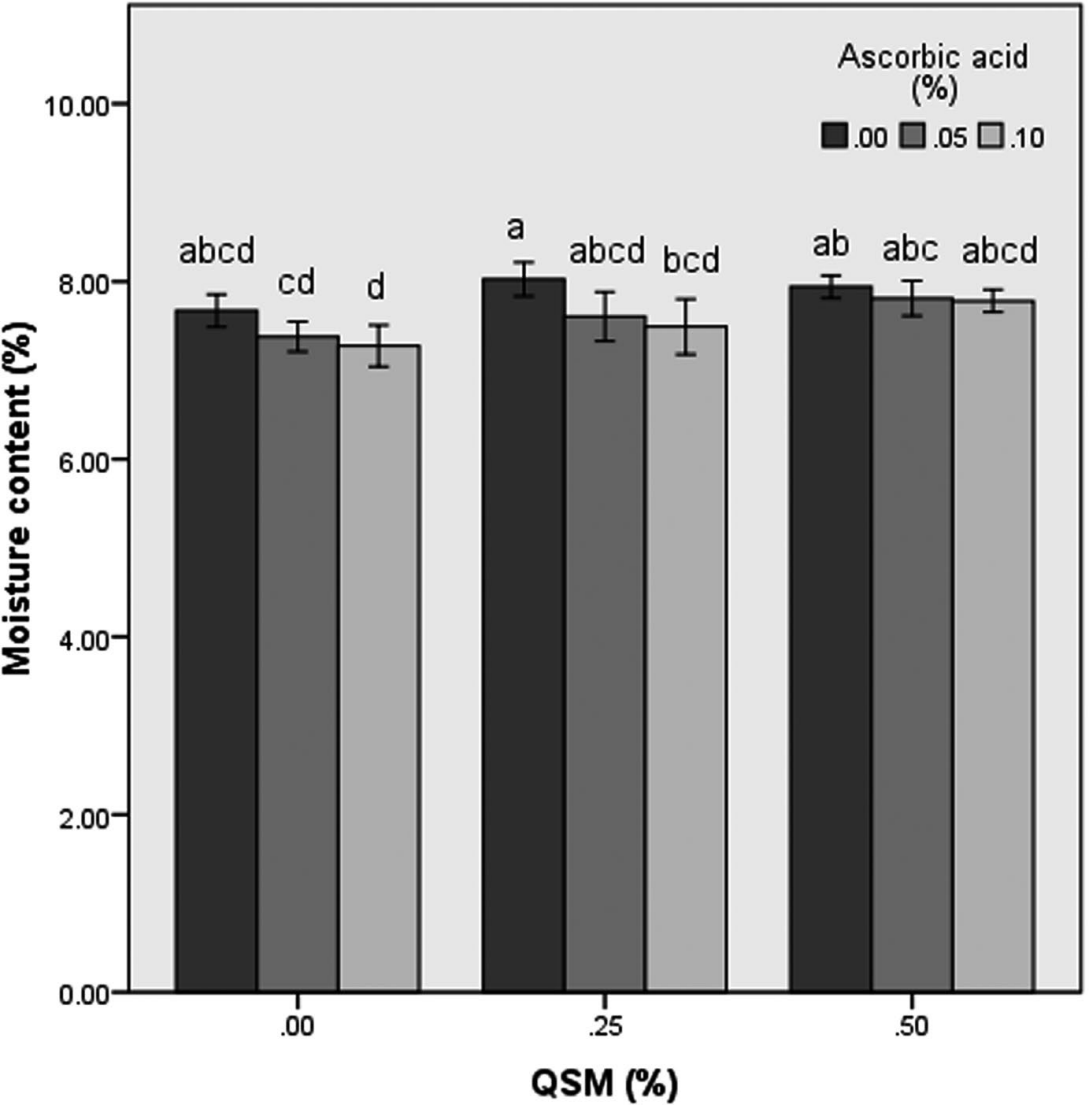
The amounts of moisture content in the control and pretreated freeze‐dried banana slices

The moisture content of the untreated dried banana slices was 7.67 ± 0.18% after freeze‐drying process (Figure 1). It is observed that moisture content was much lower in the banana chips congaing 0% mucilage compared with those containing 0.25 and 0.5% mucilage. The higher moisture in the treated banana slices could be because of the hygroscopic nature of mucilage. In fact, the mucilage as a protective layer against moisture outflow can reduce the mass transfer and by holding water and preventing moisture from being released causes viscose tissue. This phenomenon can be confirmed in microstructural analysis which shown in the next sections. Similar result was reported by Abano, Sam‐Amoah, Owusu, and Engmann (2013) and Panagiotou, Karathanos, and Maroulis (1998) in drying of mango, banana, and apple in hygroscopic sugar solutions. They showed the casehardening effect to slow down the moisture transport was increased by sugar treatment. They also reported that this solution prevents moisture outflow by creating a sticky layer around fruit slices.

The results showed that the amount of ascorbic acid was not a critical parameter in the levels of 0 to 0.25%. It can be observed that after drying process, the ascorbic acid pretreated slices showed the minimum amount of moisture content (7.38% w.b), followed by the untreated banana samples (7.67% w.b) and the mucilage dip treated banana slices (7.94% w.b).

In similar research, Abano et al. (2013) showed that treatment of ascorbic acid affects mango tissues and facilitates water dispersal when dried. Fuente‐Blanco, Sarabia, Acosta‐Aparicio, Blanco‐Blanco, and Gallego‐Juárez (2006) reported that the treatments effect on the fruit tissues so that water is more easily eliminated during the drying process. Throughout the drying process, the moisture content of the fruit slices is brought to the surface by the diffusion mechanism and then evaporated. Therefore, as it has been mentioned by Thuwapanichayanan, Prachayawarakorn, Kunwisawa, and Soponronnarit (2011), any changes that occur in the membrane which can increase the surface of mass transfer improve moisture outflow. Based on the findings of Tavakolipour and Zirijany (2014), ascorbic acid and citric acid coatings reduced drying time of banana slices up to 52% compared to the control samples in hot air and microwave dryers. In other words, moisture loss in these samples was higher than control at the specified time.

### 3.2 Antioxidant activity and total phenols

**FIGURE 2:**
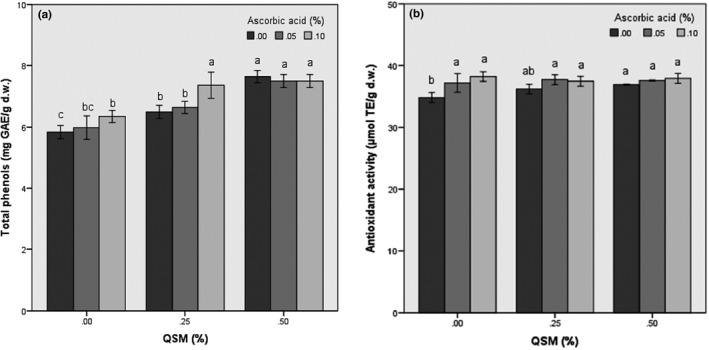
Total phenolic content (a) and antioxidant activity (b) of treated and untreated freeze‐dried banana slices

The antioxidant activity and total phenolic content of fresh‐cut banana slices before dying stage were 40.32 µmol/g and 7.88 mg GAE g^−1^ of dry basis, respectively. A decrease in TPC of dried banana chips was seen as compared to the TPC value of the fresh banana slices. This reduction in total phenols of freeze‐dried banana slices was 5.12, 19.23, and 24.35% for control (untreated), QSM pretreated, and pretreated by ascorbic acid, respectively. As shown in Figure 2, the changes in the amount of total phenols in the banana slices after the freeze‐drying process were similar to the changes in antioxidant activity. As previously established by some researchers, the antioxidant activity and phenolic content were positively correlated in fruits (Salta et al., 2010; Sharma & Rao, 2015). So, the decrease in the antioxidant activity is influenced by the decrease in the total phenolic content in banana slices.

The lower effect of freeze‐drying on the reduction of phenolic compounds compared to other methods of drying fruit slices has been reported in many studies. However, total phenolic contents of mango, hawthorn, starfruit, papaya, and sour cherry watermelon have decreased by freeze‐drying (Coklar, Akbulut, Kilinc, Yildirim, & Alhassan, 2018; Wojdylo, Figiel, Lech, Nowicka, & Oszmiański, 2014). In contrast, the amount of phenolic compounds in some studies remained constant (Shofian et al., 2011) or increased (Annegowda et al., 2014; Chang, Lin, Chang, & Liu, 2006) after the freeze‐drying phase.

The lower reduction of phenolic content of banana slices due to immersion pretreatments prior to the drying process could be due to the protective impact of these two compounds on the food product. As stated earlier by Rico, Martin‐Diana, Barat, and Barry‐Ryan (2007), ascorbic acid acts as an oxygen scavenger, eliminates molecular oxygen, and prevents the formation of compounds resulting from the polyphenol oxidase reaction. It also prevents the oxidation of the phenolic compounds of the food target by self‐oxidation and reducing the formation of quinones and also converting them to diphenols (Mayer & Harel, 1979). As illustrated by Sikora and Świeca (2018), ascorbic acid pretreatments increased potential bioaccessibility of phenolics and effectively inhibit the activity of PPO. Effect of quince seed mucilage on the antioxidant activity of freeze‐dried banana slices can be explained by the antioxidant activity of mucilage which has been stated previously (Jouki et al., 2014a). According to our previous research, the antioxidant activity of QSM at 1% concentration is equal to 29.88%. So, this active compound can be act as a protective agent for phenols and antioxidants in banana slices.

### 3.3 Color analysis and browning index

Table 1 presents the browning index and color parameters of dried banana chips. As it can be seen, the value of L* (lightness) increases from 74.29 (control) to 79.25 and 78.64 by increasing of QSM and ascorbic acid concentration in pretreatment solutions, showing that the banana becomes lighter with active treatments before drying process. The value of a* decreased from 0.072 to −2.32 and −2.83 by increasing gum and ascorbic acid concentration, respectively, revealing that a very slight green tone evolves to a red tone. The parameter b* decreased from 10.06 to 4.49 and 5.57 by increasing of concentration of dipping treatments, highlighting that pretreatments before drying process induce an decrease in the yellow intensity in banana dried slices. In this study, T7 and T3 had highest levels of QSM or ascorbic acid showed higher L* value indicating minimum browning index (5.13 and 5.32) after freeze‐drying process. These results were in agreement with the results of Farahmandfar, Mohseni, and Asnaashari (2017), which found that tragacanth and almond gums increased L* and decreased enzymatic browning in banana slices. It was found that the L * value corresponds to the browning in the banana slices. As it has been reported by McEvily et al. (1992), ascorbic acid is an effective inhibitor of enzymatic browning because of its capacity to reduce quinones to phenolic compounds before they can participate to pigment formation. Li‐Qin, Jie, Shu‐Hua, and Lai‐Hui (2009) reported that ascorbic acid had the greatest effect among the treatments used to reduce enzymatic browning in peach slices. Consistent with these results, Arias et al. (2007) reported that ascorbic acid can delay subsequent polymerization events, which are purely chemical reactions. So, by increasing the antioxidant capacity and preventing the enzyme activity by mucilage and ascorbic acid, the antioxidant properties of banana chips can be preserved and its enzymatic browning reduced. In fact, these compounds have antibrowning properties and effectively inhibit the native PPO. Antibrowning effects of QSM can be due to their antioxidant activity, which allows them to interact directly with the enzyme or react with oxidized bed molecules (Arias et al., 2007). The antioxidant activity of seed gum has been stated previously (Jouki et al., 2014b).

### 3.4 Microstructure analysis

**FIGURE 3:**
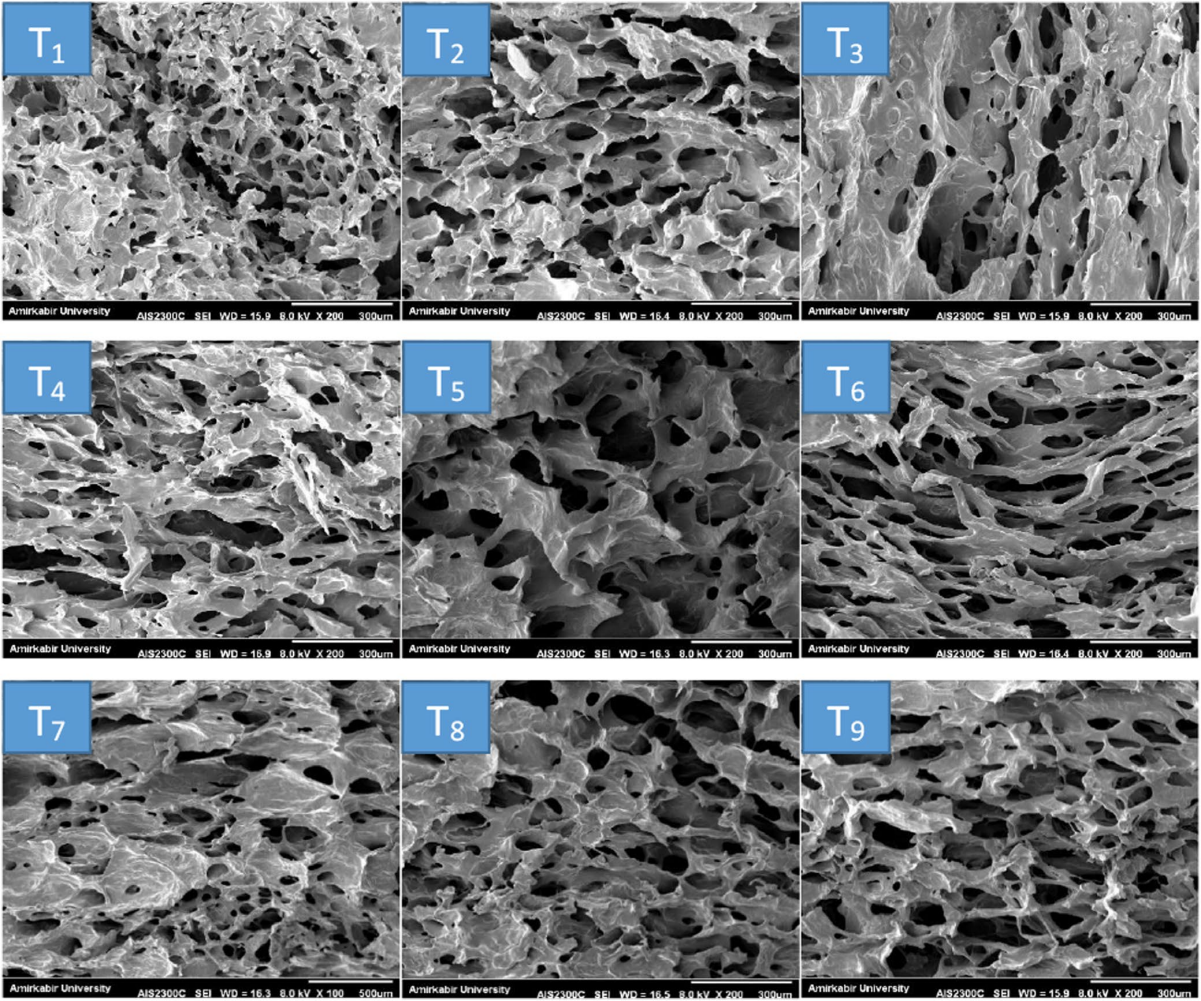
SEM of microstructure of dried banana slices after processing. Top‐to‐bottom images show increased concentration of QSM and left‐to‐right concentration of ascorbic acid

As shown in Figure 3‐T_1_, the dried banana chips had uniform and small porous structure. Dipping pretreatments with quince seed mucilage and ascorbic acid before freeze‐drying enhanced the porosity greatly and made a product with a massive and porous tissue. As it was considered in the moisture content result part, the ascorbic acid pretreatment prior to drying improved the drying conditions to eliminate more moisture from the fruit surface than the untreated banana samples. This may be due to the ascorbic acid cleanses some starch or sugar from the surface of fruit and forms more porous tissue (the right and central images). A similar trend has been reported by Pan et al. (2008) for banana slices treated with citric acid dipping before drying by infrared radiation method. They reported that immersion in citric acid treatment before infrared drying process could help to make a more porous texture of the slices and decrease the drying time. They also illustrated that the positive effects of pretreatments on the drying rate of slices occur mainly in the early stages of drying.

Immersion with mucilage solution also caused porous and massive tissue formation, although this effect was negligible and no significant changes in microstructure were seen with increasing mucilage concentration (the bottom and central images). As stated by Askari, Emam‐Djomeh, and Mousavi (2008), immersion of fresh‐cut apple slices by starch, calcium chloride, carboxymethyl cellulose, and pectin before hot air and microwave drying would enhance the porosity greatly and create a product with porous texture. However, more comprehensive studies on mass transfer and structure investigation are needed to better understand the effect of hydrocolloid dipping on drying of fresh‐cut fruit slices.

### 3.5 Sensory evaluation

**FIGURE 4:**
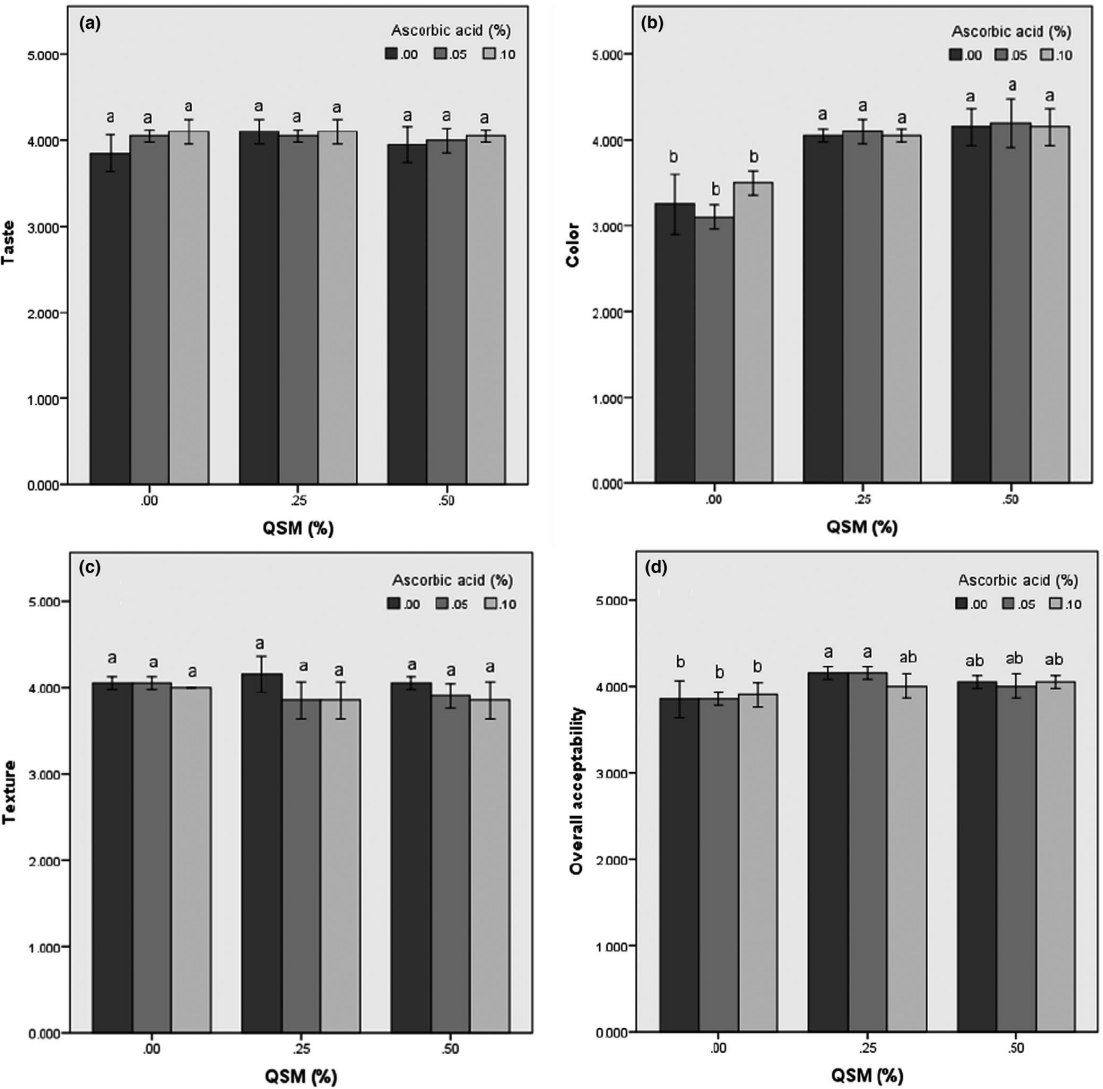
Effect of QSM and ascorbic acid concentrations on sensory scores of freeze‐dried banana slices. Taste (a), color (b), texture (c), and overall acceptability (d)

As shown in Figure 4, both ascorbic acid and QSM have no negative effect on the taste, color, texture, and overall acceptability. QSM treated slices had higher color scores (*p* < .05) than untreated and ascorbic acid treated samples (Figure 4a,b). Panelists could not distinguish significant difference between untreated dried banana slices compared to slices which treated with QSM and ascorbic acid solutions in terms of taste and texture. Statistical analysis results of the sensory evaluation showed that the use of medium concentrations of gum and ascorbic acid (0.05% ascorbic acid + 0.25% QSM) had the best effect on the sensory properties of the samples and these samples received the highest overall acceptance scores (*p* < .05). The results were in accordance with the results of the browning index and microstructure, which showed that the samples pretreated with both mucilage and ascorbic acid solutions at medium concentrations had lower browning index and higher porosity.

## 4 CONCLUSIONS

The application of both QSM and ascorbic acid treatments inhibited the enzymatic browning, thereby prevented the conversion of phenols to quinones and reduced enzymatic browning, while increased the overall acceptance of dried banana slices. The banana slices treated with both sodium chloride and gum had crisper texture and golden color than the regular freeze‐dried slices. According to the results of physicochemical, microstructural, and sensory evaluation, application of immersion treatment with 0.05% ascorbic acid + 0.25% QSM solution to inhibit enzymatic browning with preservation of qualitative properties of banana chips before drying process is suggested.

## CONFLICT OF INTEREST

The authors have declared no conflicts of interest for this article.

## ETHICAL APPROVAL

We declare no ethical issue related with this article.
